# Editorial

**Published:** 1872-08-01

**Authors:** 


MEDICAL EXAMINER.

*A Semi-Monthly Journal of Medical Sciences.*

EDITED BY

N. S. DAVIS, M. D., AND F. H. DAVIS, M. D.

Chicago, August 1st, 1872.

### EDITORIAL.

THE CHICAGO MEDICAL REGISTER AND DIRECTORY FOR 1872-3.—Published subject to the revision of the following Committee: Dr. J. W. Freer, President Rush Medical College; Dr. N. S. Davis, President Chicago Medical College; Dr. W. H. Byford, President Women's Hospital Medical College; Dr. G. C. Paoli, President Chicago Medical Society; Dr. D. B. Trimble, President Chicago Association of Physicians and Surgeons; Dr. D. W. Young, President Illinois State Medical Society.

Edited by Dr. T. Davis Fitch, Permanent Secretary Illinois State Medical Society, 296 West Monroe Street, and Dr. Norman Bridge, 267 West Monroe Street.

This volume will be issued about October 1st, 1872, and will contain about five hundred pages. It will describe the character, object, and field of operation of the hospitals, infirmaries, dispensaries, charitable institutions, and asylums of the entire State of Illinois. Also, the medical and other scientific societies and associations, with full lists of membership. It will contain, also, announcements of the schools of medicine and Pharmacy. Full directions are given respecting the means of reaching and obtaining the benefits of these various institutions. Full information is given to medical students regarding the colleges, their location, the curriculum of studies, the fees, and requirements for graduation. There is given a list of physicians, in good standing, in Chicago and vicinity, and in the larger towns of the State, with residence, office, and office hours; also, a list of dentists, regularly graduated as D. D. S. or M. D., or who are members of a dental Society; also, a list of veterinary surgeons, who are regularly educated.

The *Register and Directory* will be mailed free of postage on receipt of $2.00. The issue will be limited to 5,000 copies, and will be supplied to subscribers only. Address *Medical Register*, 296 West Monroe street, Chicago, Illinois.

It will be seen by the above circular that Doctors Fitch and Bridge have undertaken the task of preparing and publishing a Medical Register and Directory.

Such publications have been found exceedingly convenient and useful to the profession in the cities of New York, Philadelphia, Washington, etc.; and we are glad that the work has been undertaken in this city.

We think the profession can rely fully on the faithfulness and good judgment of Drs. Fitch and Bridge, and we hope they will receive promptly all the aid they need.

CHICAGO COLLEGE OF PHARMACY.—We have received the sixth annual announcement of this institution, and take pleasure in commending it to such of our readers as are interested in Pharmacy.

The faculty, fees, time, and duration of lectures are as follows:

*Faculty:* Chemistry—Prof. N. Gray Bartlett, 108 Cottage Grove Avenue; Pharmacy—Prof. James W. Mill, 133 S. Halsted Street; Materia Medica and Toxicology—Prof. D. B. Trimble, 119 Twenty-second Street; Botany—Prof. Henry H. Babcock, 11 Eighteenth Street.

*Lecture Fees:* Matriculation Ticket paid but once, $2.00; Lecture Tickets (per session), including all branches taught, $36.00; Diploma, $5.00.

The lectures of the course will be given in the college rooms, 77 Dearborn Street, commencing on the first Wednesday in October, and continuing over a period of twenty-one weeks. During the holidays there will be an intermission of one week.

The lectures will be given on Monday, Wednesday and Friday afternoons.

Prof. Bartlett will deliver the introductory address on Wednesday evening, October 2d.

